# Sphingolipids at Plasmodesmata: Structural Components and Functional Modulators

**DOI:** 10.3390/ijms23105677

**Published:** 2022-05-19

**Authors:** Yingying Zhang, Shuang Wang, Lu Wang, Xiaoyan Chang, Yongxiao Fan, Meiqing He, Dawei Yan

**Affiliations:** 1 Shanghai Key Laboratory of Protected Horticulture Technology, The Protected Horticulture Institute, Shanghai Academy of Agricultural Sciences, Shanghai 201403, China; zhangyingying2018@saas.sh.cn; 2 State Key Laboratory of Crop Stress Adaptation and Improvement, School of Life Sciences, Henan University, Kaifeng 475001, China; ws2020210@163.com (S.W.); wanglu2000209@163.com (L.W.); chang1790181776@163.com (X.C.); fanyongxiao0521@163.com (Y.F.); mqhe1030@163.com (M.H.)

**Keywords:** sphingolipid, plasmodesmata, lipidomics, microdomain, plasma membrane, signaling

## Abstract

Plasmodesmata (PD) are plant-specific channels connecting adjacent cells to mediate intercellular communication of molecules essential for plant development and defense. The typical PD are organized by the close apposition of the plasma membrane (PM), the desmotubule derived from the endoplasmic reticulum (ER), and spoke-like elements linking the two membranes. The plasmodesmal PM (PD-PM) is characterized by the formation of unique microdomains enriched with sphingolipids, sterols, and specific proteins, identified by lipidomics and proteomics. These components modulate PD to adapt to the dynamic changes of developmental processes and environmental stimuli. In this review, we focus on highlighting the functions of sphingolipid species in plasmodesmata, including membrane microdomain organization, architecture transformation, callose deposition and permeability control, and signaling regulation. We also briefly discuss the difference between sphingolipids and sterols, and we propose potential unresolved questions that are of help for further understanding the correspondence between plasmodesmal structure and function.

## 1. Introduction

The plasmodesmata (PD) channels facilitate the symplastic exchange of small molecules including photoassimilates, proteins, RNAs, hormones, and small peptides [1,2,3]. These substances flow either selectively or freely between cells satisfying the nutrient and signaling requirements during plant development and defenses [3,4,5,6,7,8,9]. The distribution and architecture of PD are adjusted according to diverse internal and external factors. The size of the largest molecule that can pass through PD is called the size exclusion limit (SEL) [10], which is regulated by the alteration of plasmodesmal composition, such as membrane lipid organization, PD-localized proteins, and the inducible accumulation of callose around PD [11,12,13]. Callose is a polysaccharide synthesized by callose synthase and degraded by β-1,3-glucanases. Its abundance is inversely correlated with the PD permeability and molecular trafficking efficiency through the channels [14,15]. The cytoskeleton is also involved in PD SEL regulation, as the disruption of actin or myosin leads to alteration of PD trafficking [16,17].

The typical structure of a PD pore consists of a cytoplasmic sleeve formed between the plasma membrane (PM) and a central desmotubule derived from the endoplasmic reticulum (ER). Microdomains composed of specific proteins and lipids are present in certain regions of the PD-PM. The “lipid raft” hypothesis proposes that nanodomain formation is promoted by the preferential association between sterols and sphingolipids creating a liquid-ordered phase within the membrane [18,19]. In a certain sense, PD are determined as a new type of membrane contact site acting as a platform for rapid molecule exchange via the cytoplasmic sleeve or fluid membrane system [20,21]. According to the morphological structure, PD can be divided into simple type and branched type (Y-, X-, V-, H-shaped) with central cavities [8,22]. In addition, the presence of cytoplasmic sleeve classifies PD as type I (no visible or a very narrow cytoplasmic sleeve) or type II (a clear cytoplasmic sleeve with visible tethering-like spokes) [23]. All these PD architectures are transformable during plant development accompanied by yet elusive changes in protein or lipid components.

**Figure 1:**
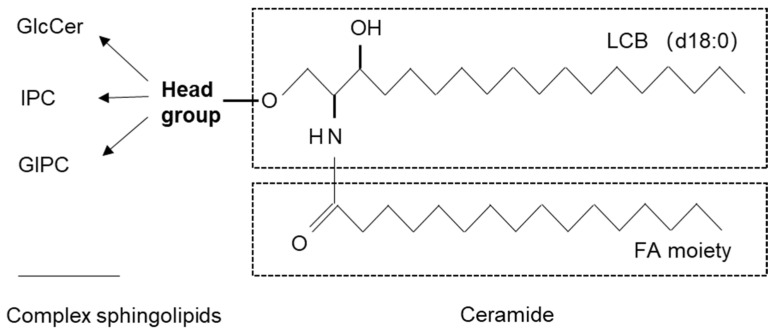
A diagram of sphingolipid structure. LCBs present as saturated form (18:0) or desaturated form (18:1) with 2/3 OH (d/t) numbers. The fatty acid (FA) chain has various carbon numbers ≥16. Ceramide is the simplest sphingolipid form. Head group indicates the substituents that are combined with ceramides to form complex sphingolipids.

Sphingolipids are essential for eukaryotic life due to their critical roles in various cellular and regulatory processes such as membrane organization, signaling, and protein sorting [24,25]. In plant cells, sphingolipids account for 40% of the PM lipid content, contributing to the cell surface activities [19,26,27]. Sphingolipids are formed by the combination of a molecule of fatty acid with a long chain base (LCB) and a head group. They are divided into different classes: ceramides (Cers), hydroxyceramides (hCers) with hydroxylated fatty acids, glucosylceramides (GlcCers) harboring a glucose head group, and glycosylinositolphosphoceramides (GIPCs) containing a head group composed of phosphoinositol with sugar residues (Figure 1). The fatty acid in complex sphingolipids often consists of a saturated or monounsaturated very-long-chain fatty acid (VLCFA) of 18 to 26 carbons in length, which can facilitate hydrophobicity, membrane leaflet interdigitation, and the gel phase transition. Notably, sphingolipids containing VLCFAs but not long-chain fatty acids (LCFAs) are reported to be critical for polar auxin transport and plant growth in *Arabidopsis* [28].

The sphingolipid metabolism in plants was well reviewed recently by Liu et al. [28]. The de novo biosynthesis of sphingolipids starts with the combining of serine with palmitoyl-CoA to produce 3-ketosphinganine by serine palmitoyl transferase (SPT), functioning as a heterodimer of LCB1 and LCB2 subunits interacting with small subunits (ssSPTs) that can increase SPT activity [29]. 3-Ketosphinganine is reduced to sphinganine (d18:0), the simplest LCB, by 3-ketodihydrosphingosine reductase [30,31]. d18:0 LCB is added a third hydroxyl group by sphingoid base hydroxylases to form t18:0 LCB [31]. Then, ceramide synthase LAG one homologs (LOHs) link d18:0 and t18:0 to a fatty acid chain to generate Cers [28]. In *Arabidopsis*, LOH1 and LOH3 preferentially participate in the synthesis of Cers with VLCFAs, while LOH2 is mainly responsible for the production of Cers carrying LCFAs [28,32]. Cers then undergo various modifications, such as hydroxylation, desaturation, and glycosylation, to produce complex sphingolipids [33]. They can also be phosphorated to ceramide 1-phosphates by ceramide kinase or degraded back to LCBs by ceramidase, probably to maintain ceramide homeostasis [34,35].

**Figure 2:**
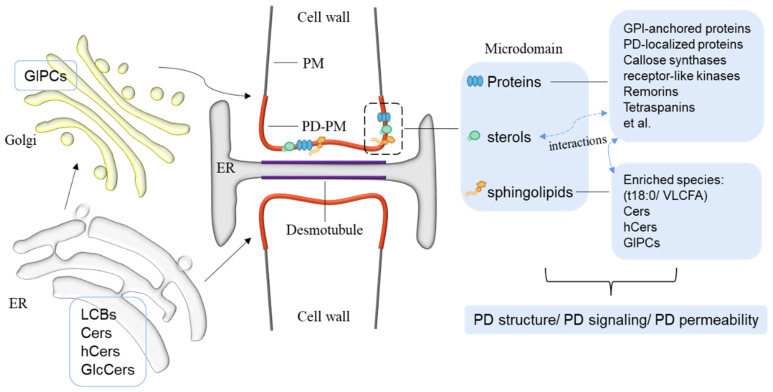
A schematic diagram of plasmodesmata architecture and components. LCBs, Cers, hCers, and GlcCers are synthesized at the ER and transported to the Golgi, where GIPCs are produced. These sphingolipid species can be sorted and transported to their destination, including the PM and PD-PM. Specific proteins, sterols, and sphingolipids, especially the t18:0/VLCFA-contained sphingolipids, are enriched in the microdomains formed at the PD-PM (red segment). They may function by interacting with each other to regulate the PD structure, signaling, or permeability, such as the experimentally verified binding of PD-LOCATED PROTEIN 5 to phytosphinganine (t18:0) [44] and potential interactions between tetraspanins and sterols [45]. The composition of the special membrane of the desmotubule (purple segment) derived from the ER is still elusive. LCBs, long-chain bases. Cers, ceramides. hCers, hydroxyceramides. GlcCers, glucosylceramides. IPCs, inositolphosphoceramides. GIPCs, glycosylinositolphosphoceramides. ER, endoplasmic reticulum. PD, plasmodesmata. PM, plasma membrane. VLCFA, very-long-chain fatty acid.

Cers and GlcCers in plants are synthesized in the ER and then transported to the Golgi where they are modified to product inositolphosphoceramides and glycosylinositolphosphoceramides (GIPCs) by inositolphosphorylceramide synthases, inositol phosphorylceramide glucuronosyltransferase1, GIPC mannosyl transferase 1, and glucosamine inositol phosphorylceramide transferase 1 [32,36,37]. After synthesis, sphingolipids may undergo intramembrane translocation, sorting, and intermembrane movement and finally localize to their targeting membranes [25] (Figure 2). In mammal cells, ceramides are transported from the ER to Golgi via ceramide transfer proteins or vesicles for the formation of complex sphingolipids, which are transported further to the PM via Golgi vesicles [38,39,40]. To date, however, very little is known about the process of sphingolipid translocation and the degradation of complex sphingolipids in plants. *Arabidopsis* ACCELERATED CELL DEATH 11 binds selectively to Cer, GlcCer, GIPC, and LCB and possesses the transfer activity for ceramide-1-phosphate and phytoceramide-1-phosphate [41,42]. Among the four glycolipid transfer proteins (GLTPs) in *Arabidopsis,* only GLTP1 can specifically transport GlcCer. GLTP2 binds to but does not transport GlcCer [43].

In this review, we summarize the progress uncovering the importance of plant sphingolipids at the plasmodesmal membrane and discuss their critical functions in plasmodesmata formation, architecture maintenance, permeability control, and signaling. However, the functional significance of sphingolipids for PD is still in the early stages of investigation. We also briefly propose the challenges and potential interesting questions. Exploring the roles and regulation mechanisms of sphingolipids may help further understand the relationship between plasmodesmal structure and function, as well as the work mode of PD. The potential findings, compared with the study of cell–cell junctions in animals, could even provide more knowledges about the various roles of sphingolipids and the evolution of cell–cell communication.

## 2. Sphingolipids Are Essential Components of Plasmodesmal Membrane

PD are seen as membrane structures as they are lined by the PM and an ER-derived desmotubule [46]. Due to the compartmentalization of PD-related function, the PM does not continue homogeneously through the whole PD channel. Currently, the elegant composition of PD remains unavailable probably due to the technical challenges in isolating a pure PD fraction, which always contains contamination from the PM or ER [11,44,47]. The use of detergent-insoluble membranes (DIMs) for biochemical characterization is questionable with regard to its consistency with the situation in vivo as the detergent treatment may lead to artificial aggregation of membrane lipids [11,48]. By optimizing the PD isolation procedure with detergent-free methods, Grison et al. obtained purified PD-enriched membrane fractions from *Arabidopsis* suspension cells without virtual contaminants from other membranes [11]. Compared with DIM fractions, their analysis revealed not only a similar lipid composition pattern but also different ratios of some lipid species [11].

Specialization of the PD fraction has been previously illustrated by the local enrichment of a specific set of membrane-associated proteins according to the published PD proteomes in *Arabidopsis*, *Populus trichocarpa*, and tobacco (*Nicotiana tabacum* L.) [49,50,51,52,53,54,55]. A ‘core PD proteome’ with 115 candidates from *Arabidopsis* was established but only 20 common candidates were identified as PD-localized proteins in the four published PD proteomes [8,52,56,57,58]. Furthermore, comparative lipidomic analysis by Grison et al. (Table 1) uncovered that the PD-PM domain is highly enriched by sterols and complex sphingolipids [11] (Figure 2). GlcCers and GIPCs, a group of highly glycosylated sphingolipids, are the two most abundant sphingolipid classes found in plant membranes. GIPCs were also the main sphingolipid classes detected in the lipid extraction from PD and PM [11]. Although the main VLCFAs detected in both the PM and the PD-enriched membrane fractions were 24:0 and h24:0, a characteristic of GIPC species in *Arabidopsis*, the PD-PM showed significant higher levels of these two classes than the PM per se [11,59]. Conversely, the PD fraction showed only about 60% enrichment of LCFA (C16-C18) versus VLCFAs (C20-C26), indicating the relative low number of glycerolipids compared with sphingolipids [11]. Moreover, the PD-PM has a higher proportion of saturated fatty acids than unsaturated fatty acids [11]. In addition to sphingolipids, phospholipids including phosphatidylethanolamine (PE), phosphatidylcholine (PC), phosphatidylserine (PS), phosphatidylinositol (PI), phosphatidic acid (PA), and phosphatidylglycerol (PG) were separated from both PD and PM fractions [11]. Notably, the PD fraction showed a higher degree of most of these phospholipids with monounsaturated and diunsaturated species but a lower degree of polyunsaturated species [11]. 

Another sphingolipid profile of purified PD and PM fractions by Liu et al. (Table 1) showed that the proportions of sphingolipid species containing C16:0, 18:0, 22:0, and 24:0 were higher, whereas C24:1, C26:0, and C26:1 were lower in the PD fraction. Among them, d18:0- and t18:0-containing C24:0 Cers, hCers, and GIPCs, but not GlcCers were enriched in the PD fraction [44]. However, the d18:1 and t18:1 LCB-based species were significantly less enriched in the PD fraction compared with the PM fraction, while the proportions of free LCBs were almost same in these two fractions [44] (Figure 2). These data indicate the potential important roles of d18:0- and t18:0-based sphingolipids in PD function. A supporting example is that the *Arabidopsis* mutant lacking both sphingolipid long-chain base 8 desaturases (SLDs) 1 and 2 that desaturate LCBs accumulated more d18:0 andt18:0 LCBs, Cers, hCers, GlcCers, and GIPCs in the PD fraction than the wildtype plants, showing decreased PD permeability in leaves [44]. Conversely, the levels of t18:0-based sphingolipids were lower in the PD fraction extracted from the SLD1 overexpression plants with increased PD conductivity [44].

To date, the composition of the ER-derived desmotubule membrane remains unclear, probably due to the lack of trustable isolation and validation methods. Given the synthesis of LCBs and Cers in ER, it would be an interesting question whether the desmotubule microdomains contain Cers or other sphingolipid species. Recently, the multiple C2 domains and transmembrane region proteins (MCTPs) have emerged as plasmodesmata-specific ER–PM tethers [58]. Their C-terminal transmembrane regions insert into the ER, and C2 domains bind to anionic lipids as PM docking sites [58]. The surface charges of the plasmodesmal PM are supposed to affect the internal membrane docking and, consequently, the cytoplasmic sleeve conductivity [58]. Notably, the component analysis of PD lipid composition is still not complete. New technologies and systematic analyses of the localization of all candidates remain necessary to build a more comprehensive map of PD-specific elements.

## 3. Sphingolipid Biosynthesis Modulates Plasmodesmal Ultrastructure

Sphingolipid biosynthesis is regulated by both key synthases and regulators [25,60,62,63]. *Arabidopsis PHLOEM UNLOADING MODULATOR* (*PLM*) encodes a novel protein involved in the sphingolipid biosynthetic pathway. The loss-of-function *plm* mutant displays a significant decrease in the levels of the trihydroxy LCBs, especially the t18:0 species, and a half reduction in the level of VLCFA-containing Cers, especially t18:0/t18:1 and C24:0/C24:1 Cers [60] (Table 1). On the other hand, the VLCFA-containing hCers and GlcCers are almost unaltered [60]. The sphingolipid profile results seem unique compared with the other reported sphingolipid biosynthetic mutants [37,45,60,61,63]. In *plm* mutants, the decrease in t18:0 does not lead to the reduction in t18:1 or any changes in phosphorated t18 LCBs; the decline in VLCFA-containing ceramides does not affect the number of LCFA-containing ceramide species [60]. PLM was proven not to be an IPCS or sphingomyelin synthase [60]. Moreover, the phylogenetic clustering indicates that PLM is plant-specific; it is proposed that PLM might function as a novel enzyme responsible for a new ceramide synthesis pathway, or it simply acts as a regulator, impairing PD architecture directly by altering PD-PM composition or indirectly by regulating PD-related signaling.

Although the sphingolipid profile was not determined using an isolated PD fraction, the absence of PLM resulted in significant changes in cellular sphingolipid levels and PD function. A fluorescent molecular movement assay showed that the loss of PLM enhances the post-SE unloading from the phloem-pole pericycle (PPP) to the endodermis due to the increased plasmodesmal conductivity of the interface between them [60]. A further study on the plasmodesmal ultrastructure found interestingly that the loss-of-function PLM mutant has no type II PD at the PPP–endodermal interface, while the wildtype Col-0 plants have an equal proportion of type I and type II PD [60]. A few intermediates, with no clear spokes but partial detachment between the two membranes, were detected in the *plm* mutant, providing evidence of unfinished architecture transformation [60]. In addition, although the *plm* mutant still had both simple and branched PD, there was no clear difference in their [60]. The compromised transition of PD from type I to type II, but not from simple to branched structures, implies the possible different mechanisms for determining these two morphogenetic processes. Given the function of PLM in sphingolipid homeostasis and the specific influence on the interface between PPP and endodermis, it is proven that VLCFA-containing sphingolipids are required for the formation and transition of PD, and it has been deduced that the requirement might be quite local.

Plasmodesmata also undergo a morphological change from simple to branched forms in some cases such as during the sink–source transition of *Arabidopsis* leaves [64]. A simple plasmodesma consisting of a single channel can be modified into a complex plasmodesma with the formation of multiple channels [65]. Until now, whether and how the cell wall-associated enzymes, signaling proteins, or sphingolipids contribute to this modification remain unclear. However, it is thought that the lipid organization in PD-PM microdomains has to be reconstructed. Thus, proteome or lipidome analysis of different types of isolated and purified PD can illuminate their precise distinct composition, as well as the underlying mechanism for their architecture modification.

## 4. Sphingolipid Metabolism Regulates Plasmodesmal Permeability

The cytoplasmic sleeve of PD has previously been well established as a continuum of symplasm between neighboring cells, and the SEL of the pores is believed to be dependent on the conductivity of the cytoplasmic sleeve [66]. Nicolas et al. then postulated a positive correlation between the spatial distribution of type I PD and enhanced trafficking [23]. Furthermore, Yan et al. provided evidence supporting this model by demonstrating that type I PD are more conductive than type II PD according to a study of *plm* mutants [60]. Therefore, sphingolipid biosynthesis modulates not only the PD architecture but also their conductivity, although the underlying mechanisms remain elusive. In addition, Nicolas et al. showed that type I PD are predominant in young root tissue, whereas type II PD are more numerous in older tissue [23]. It is, thus, worth testing the fluctuation of sphingolipid levels in different tissues and developmental stages.

As stated above, it is reasonable to propose that perturbation in sphingolipid metabolism is supposed to affect PD permeability. Seedlings treated with the inhibitors of sphingolipid metabolism, including myriocin, fumonisin B1 (FB1), dl-threo-1-phenyl-2-decanoylamino-3-morpholino-1-propanol hydrochloride (PDMP), and tricyclodecan-9-yl-xanthogenate (D609), resulted in a lower level of GlcCer and reduced PD permeability, whereas d-erythro-*N*,*N*-dimethylsphingosine (DMS) treatment enhanced the GlcCer contents and PD permeability [61]. Two glycosylphosphatidylinositol (GPI)-anchored proteins, β-1,3 glucanase2 (BG2) and PD callose-binding protein 1 (PDCB1), are mislocalized when treated by myriocin, FB1, PDMP, and D609 [61]. These results resemble the treatment by fenpropimorph, an inhibitor of sterol metabolism that affects lipid raft organization [11]. Conversely, the localization of examined PD markers was not affected when treated by DMS [61]. Accordingly, PD permeability was inversely associated with the callose deposition in these treatments. These findings suggest that sphingolipid metabolism can influence the PD permeability by modulating callose accumulation, and GlcCer level is relevant to the alteration of callose-mediated PD permeability.

PD-LOCATED PROTEIN 5 (PDLP5) is a plasmodesmal receptor-like protein localized at the central region of PD channels [67]. It positively regulates callose deposition but negatively controls PD permeability by stimulating downstream callose synthases (CalSs), for instance, CalS1 and CalS8, in *Arabidopsis* [68]. Recently, PDLP5 was found to be highly accumulated in the leaf epidermal cells of the *sld1 sld2* double mutant, where trihydroxy LCB or phytosphinganine t18:0 was elevated and the plasmodesmatal permeability was compromised [44]. Interestingly, PDPL5 was able to specifically bind to phytosphinganine (t18:0) but not unsaturated t18:1 with high affinity [44]. However, another PDLP family member PDLP1, containing a similar motif pattern to PDLP5, did not interact with t18:0 [44]. A putative sphingolipid-binding motif present in the transmembrane domain (TMD) of PDLP5, homologous to the sphingomyelin-binding motif of mammalian p24 protein, might be responsible for the binding [44,69]. This specific interaction between t18:0- or t18:0-based Cers and TMD may confer the modification of PDLP5 localization at PD [44].

Taken together, sphingolipid biosynthesis can modulate plasmodesmal permeability through either callose-dependent or callose-independent pathways. The distinct localization or compartment of callose regulators or CalSs at the PD-PM might be one of the reasons. For example, PDLP5 is supposed to be localized at the central of PD channel and PDLP1 is distributed throughout the PD membrane [68], whereas PDCBs and PDBGs are thought to localize at the PD neck regions [7]. Further characterization of the interactions between sphingolipids and CalSs or callose-associated regulators may improve and perfect the exploration of these different molecular mechanisms.

## 5. Sphingolipids Facilitate Signaling at Plasmodesmata

In the PD proteomic analysis, certain receptor-like kinases and membrane-anchored proteins were identified, implying that the PD-PM may provide a platform for their proper localization and intercellular PD-relevant signaling [52,70]. They are supposed to participate in non-cell autonomous signaling or immune responses [71,72]. Their localization at PD might be to regulate molecular trafficking while receiving apoplastic signals. For example, *Arabidopsis* LYSIN MOTIF DOMAIN-CONTAINING GPI-ANCHORED PROTEIN 2 is a PD-located, GPI-anchored receptor protein that perceives chitin and triggers PD closure; yet, the perception and signaling differ from other chitin-triggered responses such as reactive oxygen species burst or mitogen-activated protein kinase activation [73]. CLAVATA1 (CLV1) and *Arabidopsis* CRINKLY4 (ACR4) are two PM-localized receptor kinases involved in the root meristem maintenance. They function together as dimers under the control of the signaling peptide CLAVATA3/EMBRYO SURROUNDING REGION 40, whereas only the higher-order CLV1/ACR4 receptor complexes are found at the PD-PM [71]. It is deduced that the special lipid environment of the PD-PM enhances the recruitment of ACR4 to PD, and the CLV1/ACR4 multiple complexes, thus, regulate the intercellular trafficking of signaling molecules that defines root stemness by fine-tuning the PD aperture [71]. Similarly, the PD-associated signaling components can also partition to non-PD membranes for dual functions under certain conditions. PDLP1, localized at PD and PM, relocates to the membrane around the fungal infection sites with callose accumulation [74]. It is not clear yet whether lipid species are involved in this relocation.

Although plant-specific GIPCs have been reported to be involved in protein anchoring, cell-surface recognition, and signaling molecule synthesis [25,75], the precise mechanisms are still not well demonstrated. The negatively charged GIPCs are structural homologs of animal gangliosides, which can regulate Ca^2+^ homeostasis [76]. Interestingly, a recent study proved that the negatively charged GIPCs bind directly to Na^+^ on the cell surface and, thus, sense salt to trigger Ca^2+^ influx in plants [77]. This GIPC-mediated salt sensing does not resemble any known sensory system found in other organisms and may need functional partners yet unknown [77]. A study on AtACER, a ceramidase, showed that its absence promotes an increase in ceramide content and salt stress sensitivity, while overexpressing plants exhibited enhanced salt tolerance [78]. Given that the PD-PM microdomains contain a large proportion of GIPCs, these findings may imply a novel potential mechanism for PD responses under salt stress.

In addition, sphingolipids have been found acting as signaling molecules and interplaying with other signals in response to both biotic and abiotic stresses [79]. LCBs and ceramides can act as second messengers in transduction pathways. During pathogen invasion, the recognition of pathogen-associated molecular patterns by hosts can activate SPT and the released microbial mycotoxins inhibit the LOH, resulting in the disruption of LCB and ceramide contents [45,80]. Overexpressing LOH2 exhibits enhanced salicylate (SA) production and the constitutive expression of hypersensitive genes, leading to programmed cell death in *Arabidopsis* [80]. Under drought stress, *Arabidopsis* leaves accumulate more GIPCs but not ceramides; exposure to low temperature results in a decrease in the 4-hydroxy-8-sphinganine (t18:1) and total LCB contents [81,82]. Plants maintain a tight regulation of the balance between free LCBs and their phosphorylated derivatives under stress conditions. For example, the accumulation of LCBs and ceramides induces PCD, while their corresponding LCBPs and ceramide-1-phosphates inhibit cell death [83]. Taken together, it is reasonable to speculate that the sphingolipid species composing PD may participate in the stress responses of plants broadly, either as sensors or as signal communicators.

The role of sphingolipids in the defense reaction dependent on SA was proven by the enhanced levels of salicylates and resistance to pathogens in the *Arabidopsis* double mutants of *FATTY ACID HYDROXYLASE* (*FAH1* and *FAH2*) genes with depletion of complex sphingolipids [84,85], while saturated VLCFAs activated the ethylene biosynthesis and signaling pathway during cotton fiber development [85]. Furthermore, sphingolipid synthesis is also required for the membrane targeting of auxin carriers AUXIN RESISTANT 1 and PIN-FORMED 1 [28]. These studies imply the potential interactions among sphingolipids, phytohormones, and PD function.

## 6. Comparison of Sphingolipids and Sterols at Plasmodesmata

Sterols and sphingolipids are evolutionarily conserved lipid molecules acting as major plasma membrane components, and their collaboration is proposed to favor lipid microdomain formation. Similar to sphingolipids, sterols were also found to be significantly higher in PD when compared with the PM, but this enrichment seems to not show structural selectivity considering the almost similar proportion of different sterol classes within the PD-PM [11]. When treated by sterol inhibitors, plants show tissue-specific defects in callose deposition at PD. The localization of PDCB1 and PDBG2 at primary PD is altered upon the change in sterol composition [11], similar to the disruption of sphingolipid composition [61]. In cotton, suppressing *SCP2D* expression, a putative sterol carrier protein gene, led to reduced sterol contents, accumulated callose, and closed PDs at 5 through 25 days post anthesis [86]. Thus, the modification of both the sterol and the sphingolipid pools is able to interfere with callose production and, consequently, impairs the intercellular trafficking. However, the unaffected callose level in the *plm* mutant indicates the potential distinct influences on PD functionality resulting from the changes in different combinations of lipid classes at PD.

In addition to the well-known PD-localized proteins, remorin and tetraspanin, two kinds of membrane proteins that cluster in the sterol-dependent nanoscale domains of PM localize to the PD-PM microdomains [11,53,87,88]. *Arabidopsis* Remorin1.2 and 1.3 have been shown to be crucial for lipid order formation and membrane microdomain assembly, thus acting as regulators of PD aperture [13]. Tetraspanins have been reported to directly bind to cholesterol in mammalian cells [89]. Thus, whether remorins or tetraspanins bind to sterols at PD is an interesting question worthy of in-depth investigation. Sphingolipids and sterols are also supposed to interact with each other, contributing to the formation, maintenance, or compartmentalization of PD-PM microdomains, but direct evidence has not yet been reported.

## 7. Perspectives

Overall, sphingolipids, acting as key components of the plasmodesmal membrane system, participate in the regulation of plasmodesmal ultrastructure maintenance, callose deposition, signaling, and permeability control. Nevertheless, many unresolved questions are still waiting to be answered. For example, how are the sphingolipids transported to PD from their synthesis location? What signals regulate their transport when PD undergo architecture transformation? How are the specific PD-PM microdomains with a particular lipid composition established and maintained at PD and what is the diffusion barrier at the PD-PM? Do other PD-localized proteins bind to GIPC or other sphingolipids, even sterols, within the PD microdomains? In addition, sphingolipids are inevitable cellular constituents in signal transduction under biotic and abiotic stresses, such as cold, drought, salt, and microbe infection, and their biosynthesis and metabolism are altered while suffering these challenges. The influences on plasmodesmal functionality are, therefore, also worth studying during various defense responses. As proposed by González-Solís et al., the various *Arabidopsis* mutants of sphingolipid biosynthesis would be valuable resources to explore [90]. To generate a complete and accurate map of PD composition, the following methods or technologies should be further optimized: (1) PD isolation and purification for pure PD-PM, as well as PD–ER, PD–cell wall, and PD tethers; (2) multiple omics analysis for measuring the levels of numerous PD components; (3) protein or lipid labeling for validation in vivo and in vitro; (4) high-resolution microscopy for observation of PD structure and components in situ. In addition, an investigation of the precise interactions among different PD components can help us understand the comprehensive plasmodesmal organization and working mechanism.

## Figures and Tables

**Table 1:** Plant sphingolipid profiles for plasmodesmata (PD) functional study.

Material	PD Isolation	Method	Major Conclusion	Reference
Arabidopsis cultured suspension cells	Yes	LC-MS, GC-MS, Q-TOF-MS	PD membranes contain more complex sphingolipids than PM	[11]
Arabidopsis roots	No	HPLC	Sphingolipids containing VLCFA regulate plasmodesmal ultrastructure and permeability	[60]
Arabidopsis leaves	Yes	HPLC	PD membranes contain higher amounts of t18:0 lipid species than PM	[44]
Arabidopsis seedlings	No	LC-MS	Perturbation in sphingolipid metabolism alters PD permeability and GlcHcers are important for GPI-anchored PD protein localization and PD permeability control	[61]

## Data Availability

Not applicable.
